# General and Specific Genetic Polymorphism of Cytokines-Related Gene in AITD

**DOI:** 10.1155/2017/3916395

**Published:** 2017-01-04

**Authors:** Chen Xiaoheng, Mei Yizhou, He Bei, Li Huilong, Wang Xin, Hu Rui, Li Lu, Ding Zhiguo

**Affiliations:** ^1^Department of General Surgery, Dongzhimen Hospital of Beijing University of Chinese Medicine, Beijing, China; ^2^State Key Laboratory of Toxicology and Medical Countermeasures, Beijing Institute of Pharmacology and Toxicology, Beijing, China; ^3^Department of Medical Service, The Third Clinical Medical College, Beijing University of Chinese Medicine, Beijing, China

## Abstract

Autoimmune thyroid disease (AITD) shows the highest incidence among organ-specific autoimmune diseases and is the most common thyroid disease in humans, including Graves' disease (GD) and Hashimoto's thyroiditis (HT). The susceptibility to autoimmune diseases is affected by increased autoantibody levels, susceptibility gene polymorphisms, environmental factors, and psychological factors, but the pathogenesis remains unclear. Various cytokines and related genes encoding them play important roles in the development and progression of AITD. CD152, an expression product of the CTLA-4 gene, downregulates T cell activation. The A/A genotype polymorphism in the CT60 locus may reduce the production of thyroid autoantibodies. The C1858T polymorphism of the PTNP22 gene reduces the expression of its encoded LYP, which increases the risk of GD and HT. GD is an organ-specific autoimmune disease involving increased secretion of thyroid hormone, whereas HT may be associated with the destruction of thyroid gland tissue and hypothyroidism. These two diseases exhibit similar pathogenesis but opposite trends in the clinical manifestations. In this review, we focus on the structure and function of these cytokines and related genes in AITD, as well as the association of polymorphisms with susceptibility to GD and HT, and attempt to describe their differences in pathogenesis and clinical manifestations.

## 1. Introduction

Autoimmune thyroid disease (AITD) accounts for 90% of all thyroid diseases, mainly including Graves' disease (GD) and Hashimoto's thyroiditis (HT). Both diseases show similar pathological features and pathogenesis: (1) thyroid symmetric hyperplasia and thyroid lymphocyte infiltration; (2) varying number of thyroid antibodies detected in the serum; (3) family inheritance; (4) occurring in the same thyroid at the same time; and (5) GD and HT in the same patient which can be exhibited as phase transformation [1].

However, there are some differences in the pathology, clinical manifestations, and clinical outcomes of GD and HT. GD is the main cause of hyperthyroidism, accounting for more than 85% of all types of hyperthyroidism; hyperthyroidism syndrome and different degrees of goiter and exophthalmos are the most common clinical features [2]. Low thyroid parenchyma lymphocyte infiltration and simultaneously present thyrotropin receptor antibodies induce thyroid follicular cell proliferation, which eventually develops into hyperthyroidism. In HT onset, occult, early clinical symptoms are not typical because of the slow pace of development. As the disease progresses, approximately 25% of HT patients will suffer from hypothyroidism or transient hyperthyroidism, accompanied by diffuse enlargement of the thyroid. In patients with HT thyroid tissue, thyroid parenchymal lymphocyte infiltration is more serious than GD, which may cause thyroid follicular damage and ultimately become hypothyroidism [3].

AITD is a complex organ-specific autoimmune disease with risk factors that mainly include genetic factors, environmental factors, and autoimmune regulation disorders. AITD has a genetic background, and smoking, infection stress, iodine content in food, drug effects, radiation exposure, mental stress, and other factors can induce thyroid autoantibodies and autoantibodies, a process involving a variety of cytokines [4]. Under normal physiological conditions, many cytokines and immune cells precisely regulate the dynamic balance of the body's immune function. Once the body's immune balance becomes abnormal, AITD will occur [5]. Abnormal expression of a variety of cytokines such as HLA, CD152, LYP, FcRL3, CD40, and their genes will inhibit autoimmune tolerance [6].

## 2. Cytokines and Cytokine-Related Genes in AITD

### 2.1. HLA-II

HLA class II molecules are cell surface receptors that bind to antigenic peptides and present them to T cells. HLA on the short arm of chromosome 6 6p21.3 has a full length of approximately 4000 kb and consists of a group of closely linked genes, including more than 100 loci from a total of 554 alleles. HLA is currently known as the most complex and polymorphic gene in the human genome. Genetic and environmental factors play an important role in autoimmunity and increase the risk of HLA-susceptible alleles in some circumstances. HLA-II gene polymorphisms determine the diversity of HLA-II molecules. More than 70 diseases have been associated with HLA polymorphisms, and many autoimmune-related diseases are associated with HLA-II genes.

Polymorphisms in the HLA-II gene are very important for regulating immune activity. These polymorphisms determine the specificity of binding to an antigen and initiation of the immune response, as well as affecting the differentiation of T cells in the thymus. HLA also controls the secretion of cytokines and modulates the immune response by cytokine genes on haplotypes. Susceptibility alleles on HLA may also lead to GD/HT by preferentially regulating the Th2/Th1 pathways, respectively [7–10].

There are two hypotheses regarding the genetic predisposition mechanism of HLA and AITD. The structure and function of HLA itself are associated with disease development, such as the molecular modeling hypothesis, linkage disequilibrium hypothesis, autoantigen presentation hypothesis, and T cell receptor pool selection hypothesis. Another hypothesis is that other genes linked to HLA are associated with AITD. McLachlan [11] found that HLA molecules that bind to specific antigenic peptides misinterpret thyroid tissue antigens as T lymphocytes and CD4+/CD8+ T lymphocytes in the presence of environmental factors, bacteria, viruses, iodine, and stress (such as trauma). This immune response occurs through the activation of T and B lymphocytes to produce cytokines and autoantibodies, which may cause AITD. Sanjeevi et al. [12] suggested that the distal domain of the extracellular domain of HLA-II is an antigen-binding groove containing an important amino acid associated with the disease. Any changes in the amino acid at these sites can alter the nature of the interaction between HLA antigen and the T cell receptor to control the immune response to foreign and autoantigens. Barlow et al. [13] suggested that, for AITD to occur, a number of requirements must be met. Clinical symptoms of the disease are observed when environmental and genetic factors are encountered and exceed a certain theoretical threshold.

### 2.2. CTLA-4 Gene and CD152

Since Yanagawa et al. [14] first described the relationship between CTLA-4 and AITD in 1995, the correlation between CTLA-4 and the incidence of GD has been verified in a number of ethnic groups [15–18].

One of the expression products of the CTLA-4 gene is CD152, which was originally proposed by Brunet et al. [19] in 1987 and is mainly expressed on activated CD4+ CD8+ T cells. CTLA-4 is typically located in the cytoplasm and is rapidly expressed on the surface of the cell membrane 48–72 h after T cell stimulation [20]. The mechanism by which CTLA-4 downregulates the activation of T cells is as follows: CD152 expression on T cells increases in the late stage of immune activation and competes with CD28 for binding to B7. In the early stage of CD152 activation, CTLA-4 binds to the intracellular domain and mediates the negative signaling that inhibits T cell activation. CTLA-4 plays an important role in the development of a variety of autoimmune diseases. Its activation was shown to inhibit autoimmune diseases such as systemic lupus erythematosus, autoimmune glomerulonephritis, and type 1 diabetes in a variety of experimental animals [21].

Incorrect handling of CTLA-4 in the endoplasmic reticulum leads to inefficient glycosylation and reduced CTLA-4 protein expression on T cells. The reduction of CTLA-4 on T cells and variations in the CTLA-4 gene resulted in decreased CD152 expression and function, which may be related to the occurrence of AITD [22–24]. In a study on adolescent HT patients, Kouki et al. [25–29] found that CD152 expression was decreased in the HT group compared to that in the normal control group, indicating that peripheral blood T cells contained CD152 expression defects. This may abnormally activate T cells and lead to increases in thyroid autoantibodies, eventually leading to AITD. In a study by Bossowski [30, 31], CD152 expression on peripheral blood T cells increased in GD patients, which may be related to defective CD152 function caused by CTLA-4 gene polymorphisms. The increased expression of CTLA-4 on T cells is sufficiently effective to suppress the abnormal activation of T cells.

The association between CTLA-4 gene polymorphisms and GD has been demonstrated in studies worldwide. Transitions of threonine (Thr)/alanine (Ala) in the 17-codon 49-site A/G of the exon 1 leader of the CTLA-4 gene lead to errors in the handling of CTLA-4 in the endoplasmic reticulum, leading to inefficient glycosylation reaction and reduced expression of CTLA-4 protein on the T cell surface [30, 31]. The 49G allele decreased the inhibitory function of CTLA-4 on T cells. In GD patients, the AT repeat polymorphism can reduce the inhibitory effect of CTLA-4; as the length of the AT repeat sequence increases, T cell inhibitory function of CTLA-4 decreases. Genealogy scans were performed on a number of American Caucasian families with AITD, revealing thyroid autoantibodies for the individual susceptibility locus at 2q33. Compared to the control group, these families with the G (49) single-nucleotide polymorphism (SNP) of the CTLA-4 A/G genotype showed significantly increased thyroid autoantibody production [28] (*P* = 0.02).

### 2.3. PTPN22 Gene and LYP

The protein tyrosine nonreceptor type 22 (PTPN22) gene is located at 1P13.3-13.1, contains 16 exons, and encodes a 110 kDa lymphoid protein tyrosine phosphatase (LYP), a potent inhibitor of T cell activity.

LYP dephosphorylates the phosphorylated Lck, Fyn, and Zap-70 tyrosine kinases of the Src family to inactivate them by interacting with the C-terminus of Src tyrosine kinase (Csk). LYP interrupts T cell activation of signaling pathways, thereby downregulating T cell signaling [32]. LYP also suppresses T cell signaling by binding to Csk and interacts with Grb2, playing a negative regulatory role in T cell signaling [33].

LYP inhibits excessive immune responses and thus plays an extremely important role in autoimmune diseases. There are two views to illustrate this mechanism. One theory is functional acquisition. The T cell surface receptor (TCR) signal of both PTPN22 C1858T polymorphism carriers and patients was found to be weakened, but the PTPN22 expression product containing a polymorphism had stronger inhibitory effect on T cells than wild type.

In addition, the B cell surface receptors (BCR) signal is also decreased, suggesting that B cell function is also affected by gene polymorphisms. Changes in the BCR threshold may also damage B cell tolerance, and coupled with the presence of autoreactive B cells, this may lead to autoimmune diseases [34, 35]. Another paradox is the lack of gene function, which was also observed in knockout mice. The expression of LYP in PTPN22 C1858T polymorphism mice, adhesion to lymphocyte surface receptor, and immunosuppressive effect were decreased, enhancing the immune function of effector T cells, B cells, and dendritic cells [36]. The 1858 locus C→T missense mutation changes codon 620 in the LYP protein from tryptophan to tryptophan. This alters LYP function, significantly reduces the affinity to Csk, reduces the inhibition of the T cell activation signaling pathway, and may induce autoimmune diseases [37]. A recent study found that the PTPN22 1858 C/T polymorphism was associated with AITD [38], RA, SLE, and T1DM [39].

### 2.4. FcRL3

FcRL3, also known as crystallizable fragment receptor (FcR) homolog (FcRH3), is a membrane glycoprotein. The FCRL3 gene is one of five genes in the FCRL family and has obvious structural homology with the other four genes. FCRL expression is regulated by B cell differentiation, and germinal center B cells can express high levels of FCRL3 [40]. Its functional structure lies in its cytoplasmic domain, the presence of immune receptor tyrosine-activated motif (ITAM), and immune receptor tyrosine inhibitory motif (ITIM) [41]. In vitro studies have shown that tyrosine kinase syk and ZAP70 can be recruited to the ITAM region, and tyrosine phosphatases SHP-1 and SHP-2 are recruited to the ITIM region to enhance enzymatic activity and fine-tune the T cell activation signal [42].

Previous studies found that FCRL3 promoter polymorphisms are closely related to AITD and other autoimmune diseases such as SLE and RA. This polymorphism affects the expression levels of autoantibodies. The −169C susceptibility allele can bind more tightly to NF-*κ*B compared to −169T. This can significantly enhance the transcription efficiency of the gene [40]. Transfection studies revealed that the −169C allele was significantly more potent than the −169T allele, suggesting that transcriptional activity was enhanced [40]. The mechanism may increase the affinity of FCRL3 isoforms for NF-*κ*B, and NF-*κ*B enhances FCRL3 expression. Highly expressed FCRL3 promotes the production of autoantibodies. This interaction produces additional FCRL3 and autoantibodies. The FCRL3 promoter polymorphism and NF-*κ*B promoter polymorphism interact synergistically to promote the expression of FCRL3 and production of autoantibodies [43].

### 2.5. CD40

The CD40 gene is located on chromosome 20q11.2-20q13.2. CD40 is mainly expressed on the surface of antigen-presenting cells and can specifically bind to CD40L on the surface of target cells. Antigen-presenting cells present antigens to T cells to form MHC-TCR complexes. Transient upregulation of CD40L expression on the T cell surface and the synergism of CD28/B7 amplify the T cell-dependent immune response [44].

Both the membrane-bound form and free sCD40L can bind to CD40, which binds to monocyte-macrophages and secretes interleukin- (IL-) 12 from dendritic cells, while activating T cells to become helper T cells 2 (Th2), shifting the Th1/Th2 balance to the Th2 pathway. Monocyte-macrophage and dendritic cells can be induced to secrete IL-12 [45, 46], and activated T cells can be differentiated into Th2. In addition, CD40 has an important role in the differentiation and development of various stages of B cells [47]. CD40 and CD40L can also inhibit T cell function, and this negative regulation may lead to autoimmunity [48].

Immunohistochemistry and flow cytometry were used to confirm the increased expression of CD40 in thyroid follicular cells from GD patients in vivo and in vitro [49]. The level of CD40 and CD40L in the serum of patients with GD hyperthyroidism was higher than that of healthy subjects by enzyme-linked immunosorbent assay [50]. These studies showed that CD40 and AITD are closely related. CD40 in GD patients' thyroid tissue, particularly in epithelial cells, follicular cells, and fibroblasts, showed increased expression to different degrees [49]. The autoantigens of GD patients are processed by thyroid follicular epithelial cells and presented to the infiltrating T cells of the thyroid tissue to induce their activation, and CD40/CD40L upregulate the immune response during this process [51]. After T cell activation, the expression of CD40/CD40L increases on the cell surface, leading to a variety of autoimmune diseases, including GD [52, 53]. The interaction of CD40/CD40L molecules activates CD40 antigen-presenting cells, expressing FasL on the cell surface and inducing the activation of Fas+ T cells [54].

## 3. Genetic Polymorphism of Cytokine-Related Genes Associated with Both GD and HT

### 3.1. CTLA-4 Polymorphism

Polymorphisms in the promoter −318C/T, exon 1 + 49A/G in the 3′-untranslated region of exons, and CT60 in the CTLA4 gene have been confirmed to be associated with organ-specific autoimmune diseases [25, 55, 56]. The SNPs of exon 1 + 49 A/G are highly related to autoimmune endocrine diseases and are involved in a variety of autoimmune diseases including GD and HT [14, 57]. The TG gene at the 8q24 locus of the CTLA-4 gene is strongly associated with AITD. Previous studies demonstrated that amino acid substitutions occur in the SNPs of exon 33 and exons 10–12, which result in autoimmune disease in the thyroid tissue [58]. The CT60 polymorphism is one of the most closely correlated polymorphisms with autoimmune thyroid disease [59]. Studies have suggested that GD and HT are associated with CT60 [15].

By evaluating 67 newly diagnosed GD patients to identify their genotypes and autoantibodies, Zaletel et al. [60] found that for patients carrying the G genotype compared to the A/A genotype, TPOAb, the TgAb-positive rate was significantly higher. For the G allele, the median value of TPOAb was significantly higher than the other groups, confirming that the CTLA-4 exon 1G allele can produce higher levels of TPOAb and TgAb, supporting that the CTLA-4 gene plays an important role in thyroid autoantibody production. A study of HT showed that the A/A genotype produced less TPOAb and TgAb than the AG, GG genotype. These results support that the polymorphism of the CLTA-4 gene is closely related to the production of thyroid autoantibodies [61].

### 3.2. PTPN22 Polymorphism

A study conducted in 2004 showed that the PTPN22 C1858T polymorphism was associated with T1MD [62]. Subsequently, a variety of autoimmune diseases associated with T1MD, such as SLE, RA, HT, juvenile rheumatoid arthritis, vitiligo, and neutrophil-positive Wegener granulomatosis, were found. T alleles were not detected (or only a small number was detected) in other autoimmune diseases such as psoriasis, multiple sclerosis, or primary biliary cirrhosis [63–70]. These studies suggest that the PTPN22 C1858T polymorphism is a common pathogenic gene involved in autoimmune diseases.

Velaga et al. [38] evaluated a UK population and found that the PTPN22 1858T allele is the main susceptibility allele for GD. In a Polish population, PTPN22 1858T was associated with GD and negatively correlated with age at onset. The TT genotype resulted in an onset age of 20.8 years, CT genotype of 35 years, and CC genotype of at least 42 years (mean age) [71]. Tryptophan variants of the PTPN22 gene are very rare in the Asian population [72]. The association shows a significant population difference, possibly because of ancestral effects and/or the lack of susceptible variants in the population. The 1858T allele frequency decreased from the north to south in Europe, and, in central Africa and Asia, the allele was nearly nonexistent in the healthy population [73–75].

## 4. Specific Genetic Polymorphism of Cytokine-Related Gene Associated with GD

### 4.1. HLA Gene Polymorphism

In recent years, as the number of studies on HLA haplotypes has increased, it was shown that genes constituting the HLA gene cluster are highly linked. Disease-specific susceptibility genes associated with AITD are not single but are a combination of several genes. In the haplotype, each susceptible gene can independently express its genetic characteristics in order to exert its function. A series of different haplotypes are formed through the strong linkage disequilibrium in HLA genes.

In the 1990s, with the application of HLA genotyping methods, researchers carried out a more detailed analysis of susceptibility genes and found that HLA and GD were related mainly in the DQ site. In a study on Caucasians, Yanagawa et al. [76] used the PCR-sequence-specific oligonucleotide method to analyze the DQ DR allele of GD patients in the US and found that the HLA-DQA1^*∗*^0501 gene frequency was significantly higher in GD patients and positively correlated with the incidence of GD. A study on GD patients carried out in Germany and Canada also confirmed these results. In a study on Asians, Inoue et al. [77] found that Japanese patients with GD did not show a significant correlation with HLA, and Tamai [78, 79] found no significant correlation with DQ allele and GD in a Japanese population.

### 4.2. FcRL3 Gene Polymorphism

In a correlation analysis of GD and the FCRL3 SNP, rs7528684 was first identified by Kochi et al. [40] in a Japanese population. The SNP was located at position −169 of the FCRL3 gene promoter (*P* = 7.4 × 10^−5^) and was subsequently confirmed by Cappelli et al. [80] in 1059 GD patients (*P* = 0.024). Subsequently, the FCRL3-related rs3761759 SNP (tag rs7528684) detected in GD patients was also found in GD patients of Caucasian ethnicity in a study by Simmonds et al. [81]. Regression analysis showed that rs3761759 association was secondary to the FCRL3 rs11264798 and rs10489678 SNPs, and thus, FCRL3 rs11264798 and rs10489678 may affect the role of FCRL3 in the occurrence of GD. Therefore, FCRL3 SNPs can lead to GD, but the variation location for the etiological variant remains to be verified [81].

### 4.3. CD40 Gene Polymorphism

CD40 plays an important role in the development and progression of various autoimmune diseases. A study of GD and HT patients in a Chinese population found that the CD40 C/T-1 polymorphism was associated with GD risk [82]. The CD40 C/T-1 polymorphism was found significantly associated with GD in a meta-analysis of 14 studies (4214 cases and 3851 controls) and 4 studies (623 cases and 774 controls) investigating the association between CD40 C/T-1 gene polymorphism and autoimmune thyroid disease risk. Carriers of the C/C and C/T genotype had a higher risk of GD than those with the T/T genotype. However, these genotypes were not found to be associated with HT, suggesting that this is a unique feature of GD [83].

## 5. Specific Genetic Polymorphism of Cytokine-Related Gene Associated with HT

### 5.1. HLA Gene Polymorphism

Early serological analysis found that DR3, DR4, and DR5 molecules correlated with the occurrence of HT in the Caucasian population [84]. Different gene signatures associated with HT may reflect the heterogeneity of different patients. In recent years, molecular biology techniques have developed rapidly. Badenhoop et al. first used RFLP method for the analysis of HT in Caucasian patients from the UK and Canada and found that DQW7 DQB1^*∗*^0301 frequency increased. They concluded that DQW7 might be an immunogenetic marker on susceptible HLA haplotypes [85]. Shi et al. [86] found that the frequency of DQA1^*∗*^0301 DQB1^*∗*^0201 in Canadian patients with HT was significantly increased by PCR-sequence-specific oligonucleotide, and the susceptibility of HT might be through DQA1^*∗*^0301/DR4 DQB1^*∗*^0201/DR3.

## 6. Conclusion and Prospects

The pathogenesis of AITD is not completely clear. In the natural course of AITD, cytokines play a critical role, particularly in breaking autoimmune tolerance, autoantigen presentation, T and B lymphocyte activation, autoantibody production, and inhibition of autoimmunity. Therefore, changes in the expression of these cytokines are important pathogenic factors of AITD.

Although the mechanism is still not entirely clear, the occurrence of GD and HT has the same cytokine-related genes abnormalities. These aberrantly expressed genes may indicate a common occurrence in AITD. The PT1822 C1858T polymorphism was found to increase the risk of GD and HT [38]. Because C1858T can produce LYP, one of the AITD-related cytokines, expression decreases and the effect of inhibition of T lymphocytes is weakened, leading to autoimmune diseases. Therefore, PTPN22 may be a key gene in the breaking of immune tolerance. As inhibitory factors, CTLA-4 gene polymorphism [25, 55, 56, 87] and A/A genotype [60] expression may reduce the production of TPOAb and TgAb, leading to the onset of AITD. These cytokine-associated gene polymorphisms directly affect cytokine expression levels to break immune tolerance, causing specific organ damage. In future studies, the role of PTPN22 in the process of autoimmune tolerance breaking during the initial phase of autoimmune disease as well as the negative immunoregulatory effect of the CTLA-4 gene should be determined to reveal the common mechanisms of these diseases.

GD and HT are the same type of diseases, but their clinical manifestations and the final outcomes are very different. This suggests that GD and HT also have their own specific pathogenic mechanisms in addition to the cooccurring mechanisms of AITD. Although both GD and HT exhibit autoimmune tolerance breaking and autoantigen presentation, the expressions of various cytokines such as HLA-II molecules, CD152, FcRL3 molecules, and CD40 are different and are closely related to HLA-II, CTLA-4e, FcRL3, and CD40 gene polymorphisms. FcRL3 promoter polymorphism and CD40/CD40L high expression will lead to increased autoantibodies. This will only lead to the occurrence of GD while the aberrant expression of other genes will only induce HT. This apparent difference in cytokines and cytokine-related genes may explain the differences in the pathogenesis of GD and HT during disease progression and their underlying clinical manifestations. Additional studies on the differential genes and cytokines may reveal the pathogenesis of GD and HT, providing a foundation for the development of specific treatments.
